# 3D Bioprinting of Cartilage for Orthopedic Surgeons: Reading between the Lines

**DOI:** 10.3389/fsurg.2015.00039

**Published:** 2015-08-13

**Authors:** Claudia Di Bella, Amanda Fosang, Davide M. Donati, Gordon G. Wallace, Peter F. M. Choong

**Affiliations:** ^1^Department of Orthopaedic, St Vincent’s Hospital, Melbourne, VIC, Australia; ^2^Department of Surgery, University of Melbourne, Melbourne, VIC, Australia; ^3^Murdoch Childrens Research Institute, University of Melbourne, Parkville, VIC, Australia; ^4^Unit of Orthopaedic Pathology and Osteoarticular Tissue Regeneration, Rizzoli Orthopaedic Institute, Bologna, Italy; ^5^ARC Centre of Excellence for Electromaterials Science, AIIM Facility, Intelligent Polymer Research Institute, University of Wollongong, Wollongong, NSW, Australia

**Keywords:** bioprinting, osteochondral injuries, cartilage, addictive manufacturing, tissue engineering

## Abstract

Chondral and osteochondral lesions represent one of the most challenging and frustrating scenarios for the orthopedic surgeon and for the patient. The lack of therapeutic strategies capable to reconstitute the function and structure of hyaline cartilage and to halt the progression toward osteoarthritis has brought clinicians and scientists together, to investigate the potential role of tissue engineering as a viable alternative to current treatment modalities. In particular, the role of bioprinting is emerging as an innovative technology that allows for the creation of organized 3D tissue constructs via a “layer-by-layer” deposition process. This process also has the capability to combine cells and biomaterials in an ordered and predetermined way. Here, we review the recent advances in cartilage bioprinting and we identify the current challenges and the directions for future developments in cartilage regeneration.

## Introduction

Orthopedic surgeons commonly face clinical and surgical challenges for which current therapeutic strategies are not able to provide a satisfactory result. An example are young patients with large osteochondral defects due to injury or osteochondritis dissecans, which represents a difficult and frustrating clinical scenario for both the patient and the surgeon. Previous hyaline cartilage damage has been reported to predispose individuals to osteoarthritis, possibly due to the limited capacity of hyaline cartilage to repair itself (1).

The inability to halt degenerative changes in the articular surface in patients with chondral and osteochondral lesions has brought scientists, clinicians, and surgeons together to tackle the difficulties in cartilage tissue engineering. The goal of such collaboration is to produce mature hyaline cartilage that can maintain its physical and functional properties in the long term, without accelerated degeneration that may lead to arthritic changes.

Microfractures, mosaicplasty, and osteochondral allografts are the most common solutions for a young patient with an osteochondral defect. Options like membrane autologous chondrocyte implantation (MACI) and other autologous chondrocytes implantation techniques have failed to demonstrate sufficient superiority over the former techniques (2–6) leading to a loss of support from important jurisdictional advisory committees because of the large cost differential (7).

Tissue engineering has the potential to address the issue of osteoarticular loss and may provide a viable alternative to current treatment modalities. For example, established *in vitro* and *in vivo* tissue-engineering techniques have successfully led to the creation of living cartilage (8–11) and bone (12, 13).

The capability to re-growth living tissue at the core of their complexity remains a major challenge due to the differences in cell types, matrix components, and organization (14), and this is particularly true for hyaline cartilage regeneration. Tissue engineering can yield three-dimensional (3D) tissue-like constructs, which are known to be important for organ development and in addition these can serve as “experimental platforms for biological studies and drug screening, and as implants for clinical application” (15).

Bioprinting can be defined as an “innovative technology that allows for the generation of organized 3D tissue constructs via a layer-by-layer deposition process that combines cells and biomaterials in an ordered and predetermined way” (16, 17). Bioprinting of scaffolds and cells is emerging as an important way of recreating the microphysical environment and the relationship between cells, their matrix and local anatomy. There is a great variety of 3D printing techniques, each with pros and cons and with particular indications to specific tissues.

The goal of this review is to focus on recent advances in cartilage bioprinting and to identify the current challenges and the directions for future developments in cartilage regeneration.

## Cartilage: Why is it Difficult to Recreate the Perfect Articular Surface

Without blood vessels, nerves, and lymphatics, and with only one type of cells (18, 19), mature hyaline cartilage appears to be easy to create in laboratory. However, these characteristics also mean that cartilage injuries cannot heal spontaneously, and that any type of repair will be characterized by fibrocartilage, which represents a “scar-type” tissue (20, 21). This tissue lacks the properties that make hyaline cartilage so unique including its resistance to shear, compression, and load, thus leading to degenerative changes and arthritis (22).

Despite its simple appearance, cartilage is, in fact, a tissue that shows great heterogeneity, and is characterized by a composition that exhibits differences depending on the depth of the tissue. Articular cartilage can be divided into three zones: the “superficial zone” (SZ) represents the top 10–20% (area in contact with synovial fluid); just deep to it, the “middle zone” (MZ) represents the next 40–60% of the cartilage, and, finally, the “deep zone” (DZ) the bottom 30–40%, which then is in direct contact with the subchondral bone. The SZ is characterized by the highest cell density, the lowest amount of glycosaminoglycans (GAGs) (23), and the lowest biosynthetic activity (24). Moving deeper from the SZ, there is a progressive decrease in cell density and an increase in the amount of GAGs (23), which results in the greatest amount of GAGs and the lowest cell density in the DZ. A high concentration of GAGs determines an increase in the compressive modulus of the tissue, which therefore is at its peak in the DZ (25).

**Figure 1:**
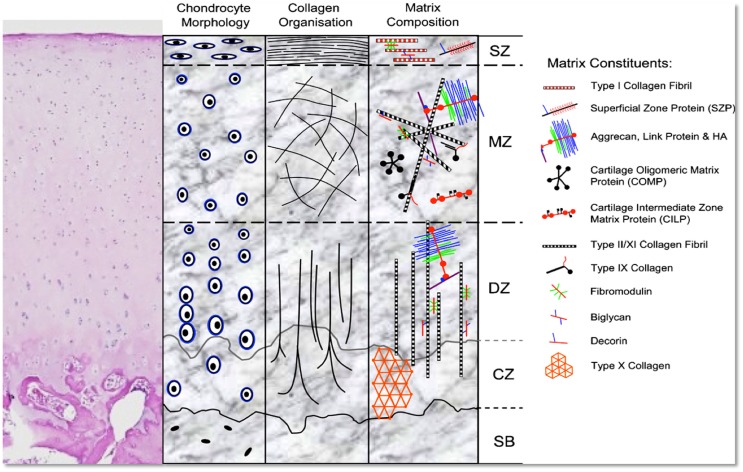
**H&E stain and schematic representation of hyaline cartilage morphology and structure**. SZ, superficial zone; MZ, middle zone; DZ, deep zone; CZ, calcified zone; SB, subchondral bone. Picture used with permission obtained from *J Cytochem Biochem*.

With regards to cell distribution and morphology, chondrocytes in the different zones differ. In the SZ, cells are small and flattened, while in DZ, cells are larger and round (26). Furthermore, collagen fiber alignment shows a very characteristic “arcade-like structure” (27): collagen fibers, in fact, originate from the calcified cartilage in a direction perpendicular to the joint surface, and then change their orientation in the MZ to become parallel to the articular surface in the superficial layer. This specific disposition of collagen fibers, together with the distribution of the proteoglycan aggregates between the fibrils, provides the tissue with unique biomechanical characteristics, which combines compressive stiffness, resilience, and shear resistance. Additionally, different types of proteins are present in the articular cartilage, and their secretion and prevalence differs among zones; in the SZ, the most represented proteins are clusterin (28, 29), proteoglycan-4 (PRG4), also known as superficial zone protein (SZP) or Lubricin (30) and Del-1 (31), while in MZ, cartilage intermediate layer protein (CILP) (32, 33) is at its peak. On the other hand, cartilage oligomeric matrix protein (COMP) is mainly seen in MZ and DZ (34, 35). The specific distribution of these proteins probably contributes to the “zone-specific functionality” of the cartilage (Figure 1).

It seems clear that the vast heterogeneity of articular cartilage makes it a tissue much more complex than initially thought to engineer *in vitro*.

A recent review on cartilage regeneration using zonal chondrocyte subpopulations has concluded that the attempted restoration of the native tissue organization of articular cartilage has had very limited results to date (36). It is well-known that the topographical heterogeneity in biochemical and structural ECM characteristics of articular cartilage is mainly due to the influence of biomechanical load and the microenvironment (37); therefore, some authors question strategies based on the use of zonally harvested cells, considering these as overcomplicated and potentially even inherently ineffective.

On the other hand, an approach that is based on the use of a single cell source coupled with the adequate biochemical and/or biomechanical stimuli can prove to be more effective and simpler.

Another promising approach for tissue engineering is the combination of a structure that shows biomechanical characteristics similar to the natural environment, in order to create a similar force dissipation pattern using “juvenile” cells, such as mesenchymal stem cells (MSCs) or chondroblasts. This approach seems feasible, and, in fact, it has been shown that combining different biomaterials with a “smart scaffold design” can potentially affect the deposition of extracellular matrix (ECM) by influencing cell alignment (38). For the fabrication of such complex multiple-material structures, advanced manufacturing techniques (Bioprinting) have been shown to be useful (39–41) and, potentially, the way of the future.

## Types of Bioprinting: What Works for Osteoarticular Tissues

Considering the inherent shortcomings of conventional scaffold-based tissue repair, a new bio-fabrication approach, termed “three-dimensional (3D) bioprinting,” has been introduced in regenerative medicine (16). Differing from “subtractive manufacturing” traditionally used to create scaffolds (i.e., creating a shape by chipping away parts from a large block), the new emerging technology is “additive manufacturing” (AM), which involves the ability to create objects from the bottom-up. A 3D printer is therefore a “computer controlled robotic system that creates three-dimensional objects through the layer-by-layer addition of material” (42). Using 3D printing techniques, the time required to modify a test product is dramatically reduced. From this, the term “Rapid Prototyping” is used.

Technological advances in the fields of automation, miniaturization, and computer-aided design and machining have led to the development of bioprinting (16–40). Applications of rapid prototyping in regenerative medicine allow tissue engineers to precisely control the scaffold structure, and hence, guide cells to form a functional tissue (43, 44).

With the boom of 3D bioprinting and new engineering technologies to create scaffolds of different materials and shape, there has been a wide development of printers and machines. Several AM technologies that allow the fabrication of customized parts and devices with geometrically complex structures have been applied in the field of bio-fabrication (45). These include fused deposition modeling (FDM) (46, 47), pneumatic extrusion printing, stereolithography (48–50), extrusion printing gels (51), inkjet printing (52–55), and selective laser sintering (SLS) (56, 57). Each of these methods has advantages and disadvantages; however, a detailed discussion of these is beyond the scope of this review. With regards to cartilage regeneration, hydrogel-based scaffolds are the main materials used given their inherent compatibility with chondral tissue; therefore, inkjet and pneumatic extrusion printers are the most commonly used machines in this field of tissue engineering.

With advances in AM-based printing technologies, a certain degree of material specificity can be also engineered, and this includes highly ordered interconnected porous polymer network structure (58). Moreover, the ability to print cells together with the scaffold can facilitate the production of biomaterial that can have characteristics similar to native tissue. The development of such a technology able to combine the deposition of specific cell types with the simultaneous printing of biomaterials can, potentially, be useful in the creation of cartilaginous tissue with different zonal distribution (59).

All the printing techniques described above have been used to print cells, and, although with some differences, all have demonstrated to be safe and reliable with regards to cells survival and proliferation.

## Bioprinting Cartilage

Hydrogels are defined as “water-swellable, yet water-insoluble, cross-linked networks” that can provide multiple advantages in tissue engineering as cell carriers for the creation of a multiple tissues. The 3D environment that they provide is able to maintain a high-water content, which resembles biological tissues and, therefore, facilitates cell proliferation (60). There are a multitude of natural polymers (i.e., collagen, chitosan, hyaluronic (HA) acid, silk proteins, gelatin and alginates) that are widely used as hydrogel materials for tissue-engineering applications, in particular, for cartilage tissue engineering (61, 62). Biocompatible hydrogels have the ability to induce a phase change from liquid to (semi-)solid by crosslinking (63), and for this reason these materials show high potential for 3-D bioprinting. Crosslinking can be induced chemically (e.g., Ca^2+^ to cross link alginate), thermally, or using UV or visible light with the addition of appropriate initiators.

In cartilage bioprinting, it has been shown that “chondrocytes and stem cells encapsulated within alginate hydrogels remain viable and metabolically active” (64). The main limitation of hydrogels for tissue engineering is their inability to maintain a uniform 3D structure. To overcome this problem, hydrogels may be coupled with synthetic biomaterials, such as poly-glycolic acids (PGA), polycaprolactone (PCL), methacrylate, hydroxyapatite, and others. A combination of hydrogel, in the form of alginate–gelatin, and hydroxyapatite can be used to print stable 3D constructs for bone regeneration, and this combination also allows living human mesenchymal stem cells (hMSCs) to be added in the bioink. This approach has shown that, after 3 days of *in vitro* culture, cell viability remains high despite the printing and crosslinking processes (65).

Hyaluronic acid (HA) is an essential component of the cartilage ECM and “its structural and biological properties mediate cellular signaling, wound repair, morphogenesis, and matrix organization” (66). Recently, HA has, in fact, been used more and more often as an important “building block” for the creation of new biomaterials in cell therapy approaches, three-dimensional (3D) cell culture, and tissue engineering (67–69). HA has been widely used as hydrogel for cartilage regeneration, as an ECM-mimetic hydrogel with good results (70–72).

Many studies have used materials, such as PCL or polylactic acid (PLA), together with hydrogels for the creation of a printable material, compatible with cartilage cells. PCL was successfully used for the creation of 3D-printed scaffolds using a “layer-by-layer” deposition strategy and coupled with “chondrocyte cell-encapsulated alginate hydrogel” (73). This study showed the formation and synthesis of cartilaginous matrix without any adverse tissue response.

In another study, PCL fibers were deposited using electrospinning techniques, which were alternated with inkjet printing of chondrocytes (derived from rabbits) and suspended in a fibrin–collagen hydrogel. This strategy was used to fabricate a tissue construct of 1 mm thickness, made of five-layers of material combined together. The authors show that this fabricated constructs allowed the formation of cartilage-like tissues both *in vitro* and *in vivo*, and this was demonstrated by the deposition of type II collagen and GAGs (74).

Methacrylate containing materials are commonly used with hydrogels for the regeneration of cartilage. An example is the use of Poly(ethylene glycol) dimethacrylate (PEGDMA) printed together with human chondrocytes to repair defects in osteochondral plugs (3D biopaper) in “layer-by-layer” assembly (75). In this study, an osteochondral defect was created *in vitro* in the center of an osteochondral plug, and using inkjet printing methods, PEGDMA and chondrocytes were printed within the defect. The authors demonstrated that delivering chondrocytes and biomaterial scaffolds to precise target locations in a 3D for zonal cartilage engineering is a feasible strategy of fabricating cartilage structures with anatomic characteristics.

Cells can also be printed by encapsulating them into micro-carriers. Levato and colleagues have shown that cell-laden PLA micro-carriers can be encapsulated in gelatin methacrylamide-gellan gum bioinks, and using this approach they have fabricated a bi-layered tissue models that included not only the cartilage tissue but also the bone compartment (14).

Recently, Kesti has shown that a scaffold made of the thermoresponsive polymer poly(*N*-isopropylacrylamide) grafted hyaluronan (HA-pNIPAAM) with methacrylated hyaluronan (HAMA) could be used for creating cartilage *in vitro*. The HAMA-HA-pNIPAAM was cross-linked using UV light. Bovine chondrocytes were cultured on top the scaffold and showed 98% viability after 7 days of culture, demonstrating that the combination of hydrogel and HAMA was not toxic and that UV light does not affect cells viability (76). However, when cells were printed in the context of the gel, the survival rate was severely affected (77). Cells within the gel tend to show a limited interaction between each other, and this could be explained by the nature of alginate, which does not allow for strong cell–cell communication. Thus, although there were some successful reports about bioprinting of cell-printed structure, great concern remains regarding the minimal cells–material interactions and inferior tissue formation compared to tissues that have not been printed and cross-linked (78).

It would seem ideal if cells are provided the natural microenvironment that exhibits similar characteristics to their original tissue, such as decellularized extracellular matrix (dECM). The recapitulation of ECM, in fact, has become the focus of cartilage engineering in recent years. It is hypothesized that chondrocytes would change their function and morphology based on the ECM, therefore being able to provide the appropriate ECM structure is now considered paramount in cartilage tissue engineering. So far, however, the complexity of natural ECM has not been able to the replicated by the majority of the matrix materials used for bioprinting and thus these materials have not demonstrated yet their ability to reconstitute the intrinsic cellular morphologies and functions of articular cartilage. Recently, a bioprinting method for printing of cell-laden structure with novel decellularized ECM (dECM) bioink capable of providing an optimized microenvironment conducive to the growth of 3D structured tissue has been described (78). In this study, printed cell dECM constructs revealed high levels of cell viability, differential lineage commitment, and ECM formation. With this approach, the authors were able to generate a tissue *in vitro* that had analog characteristics of the original tissue, with either adipogenic or chondrogenic potential, based on the type of dECM used.

Finally, it has been proposed that Bio-fabrication of tissues can be done without the use of a 3D scaffold. Laser printing of stem cells for bio-fabrication of Scaffold-Free Autologous Grafts for bone and cartilage tissue engineering has been shown by Gruene et al. to be a reliable way of producing cartilage *in vitro* (79). Using a natural hydrogel consisting of plasma and alginate, stem cells have been successfully printed *in vitro* and differentiated toward mature cartilage and bone.

## Current Challenges

Although research in cartilage bioprinting is growing exponentially, there is still a lack of *in vivo* studies that can ascertain the capability of the printed material to regenerate hyaline cartilage. In particular, the big challenge remains the long-term stability of the engineered tissue. So far, no studies have demonstrated the superiority of these techniques to the currently used clinical strategies; and therefore, we are still far away from the use of bioprinting in clinic.

One of the difficulties is to obtain ethical approval for the harvest and expansion of stem cells in laboratory and, subsequently, their use in surgery. The phrase “bench-to-bedside” is commonly used to describe the translation of basic discoveries, such as those on stem cells to the clinic for therapeutic use in human patients. This is still a very difficult obstacle to overtake before the discoveries made in laboratory can be safely and successfully translated in human patients (80, 81).

Another challenge is the matching of the bench-based printed material to the operating room. Despite the advances in 3D anatomic reconstructions, the *in vitro* printed material will not be able to perfectly match the defect that needs to be regenerated. The current *in vivo* studies are based on man-made regular defects that can be filled with a scaffold made of the exact shape and dimension. In clinics, however, this is not the case, and although more defined defects can be created (such as the ones made for mosaicplasty), this is not ideal as it further increases the area that needs to be repaired. Printers are big machines connected with highly sophisticated computers, and at this stage, the only solution is to obtain the material in laboratory and subsequently transfer it to the patient. “*In situ*” bioprinting has been performed by Cohen and colleagues, who used an explanted articular surface from a calf and, by holding it on a support, printed “*ex vivo*” alginate hydrogel for bone and cartilage repair (82). Even in this case, however, the machine used for the printing is too cumbersome to be used in an operating room. There is the need, therefore, to create a printing system that can be used “live” during the surgical procedure, directly by the surgeon. This could represent the future for tissue engineering using bioprinting techniques in cartilage regeneration, as it would avoid some laboratory-based passages, which would represent more ethical challenges. Using a single direct approach, also, the need for two (or more) surgical interventions will be eliminated, with better compliance for the patient and a quicker recovery time.

Overall, the possibilities that bioprinting brings to tissue engineering are endless, and for the scientific community this is a very exciting time. There is still some time to wait for these technologies to be available to the surgeons, but the findings so far are very promising.

## Conflict of Interest Statement

The authors declare that the research was conducted in the absence of any commercial or financial relationships that could be construed as a potential conflict of interest.
